# Controlling the nutritional status score: a new tool for predicting postoperative mortality in patients with infrarenal abdominal aortic aneurysm treated with endovascular aneurysm repair

**DOI:** 10.3389/fnut.2024.1351797

**Published:** 2024-05-01

**Authors:** Sheng-Lin Ye, Tian-Ze Xu, Chuang Wang, Kang Han, Xu-Dong Jiang, Tao Tang, Bin Song, Xiao-Long Du, Nan Hu, Xiao-Qiang Li

**Affiliations:** Department of Vascular Surgery, Nanjing Drum Tower Hospital, The Affiliated Hospital of Nanjing University Medical School, Nanjing, China

**Keywords:** malnutrition, controlling nutritional status score, abdominal aortic aneurysm, endovascular aneurysm repair, midterm mortality

## Abstract

**Background:**

AAA is a fatal condition that commonly occurs during vascular surgery. Nutritional status exerts a significant influence on the prognosis of various pathological conditions Scores from the CONUT screening tool have been shown to predict outcomes of certain malignancies and chronic diseases. However, the ramifications of nutritional status on AAA patients undergoing EVAR have not been elucidated in prior studies. In this study, we aimed to elucidate the correlation between CONUT scores and postoperative prognostic outcomes in patients with AAA undergoing EVAR.

**Methods:**

This was a retrospective review of 177 AAA patients treated with EVAR from June 2018 to November 2019 in a single center. Patient characteristics, CONUT scores, and postoperative status were collected. These patients were stratified into groups A and B according to CONUT scores. Subsequently, a comparative analysis of the baseline characteristics between the two cohorts was conducted. Cox proportional hazards and logistic regression analyses were employed to identify the autonomous predictors of mid-term mortality and complications, respectively.

**Results:**

Compared with group A, patients in group B had higher midterm mortality (*p* < 0.001). Univariate analysis showed that CONUT scores; respiratory diseases; stent types; preoperative Hb, CRP, PT, and Fb levels were risk factors for death. Multivariate analysis confirmed that CONUT score [HR, 1.276; 95% CI, 1.029–1.584; *p* = 0.027] was an independent risk factor for mortality. Logistic regression analysis showed that prior arterial disease, smoking, and D-dimer levels were risk factors, although multivariate analysis showed smoking (OR, 3.492; 95% CI, 1.426–8.553; *p* = 0.006) was an independent risk factor. Kaplan–Meier curves showed that patients in group B had shorter mid-term survival than those in group A (log-rank *p* < 0.001).

**Conclusion:**

Malnutrition was strongly associated with mid-term mortality in patients with infrarenal AAA treated with EVAR.

## Introduction

Abdominal aortic aneurysm (AAA) is characterized by a focal, enduring pathological expansion of the abdominal aorta, predominantly manifesting in the infrarenal abdominal aortic region, with a minority of cases (5%) occurring proximal to the renal artery (1, 2). The population-based incidence of AAA detected by screening ultrasound is 4–7% in men and 1–2% in women older than 65 years (3). The etiology of AAA is related to age, male sex, smoking, and positive family history (4, 5). Most patients with AAA have no obvious symptoms, but as the size of the aneurysm gradually increases, the risk of its rupture increases significantly (6). The occurrence of ruptured abdominal aortic aneurysms (rAAAs) poses a formidable challenge in vascular surgery, associated with a pronounced mortality risk (1, 7) and a high incidence rate of 81% according to a report from the United States Preventive Services Task Force (8).

At present, there are two main treatments for AAA, namely open surgical repair (OSR) and interventional surgery. Traditionally, OSR was considered the standard of care for AAA; nevertheless, endovascular aneurysm repair (EVAR) is progressively gaining preference due to its minimally invasive nature and accelerated recuperative trajectory. The Society for Vascular Surgery guidelines recommend EVAR for the treatment of infrarenal AAA (9).

The assessment of nutritional status has recently become a key decision point for surgeons to assess the preoperative physical condition of patients, especially elderly patients. The Controlling Nutritional Status (CONUT) score is an objective screening tool to assess nutritional status (10). The CONUT score is calculated based on serum albumin, lymphocyte count, and total cholesterol, and these clinical data are easy to collect. CONUT scores have been found to be a prognostic factor in patients with certain malignancies or chronic diseases. CONUT scores can also be used as a prognostic indicator for patients with end-stage liver disease (11) and acute heart failure (12). They are also associated with disease activity in patients with lupus nephritis (13). In addition, they have been shown to be associated with prognosis and treatment response in patients with cancer (14–18). Most patients with AAA are older and hypertensive, which is consistent with the finding that CONUT scores are associated with survival rates in hospitalized older patients (19) and hypertensive patients (20). Therefore, it is reasonable to believe that the CONUT score is closely related to the prognosis and mortality of patients with AAA.

The aim of this investigation was to retrospectively study the clinical data and the follow-up results of patients with infrarenal AAA who underwent EVAR and to analyze the relationship between the CONUT score and the midterm prognosis of patients with infrarenal AAA, so as to construct a prognostic model based on the CONUT score and to evaluate its predictive ability.

## Methods

### Study cohort

This was a single-center retrospective review, focusing on patients diagnosed with infrarenal AAA undergoing EVAR. The research protocol adhered to the ethical principles outlined in the Declaration of Helsinki and received approval from the Ethics Committee of Nanjing Drum Tower Hospital, affiliated with Nanjing University School, under the ethical board reference number 2021-354-02. Prior to the surgical procedure, all patients provided written informed consent.

A total of 228 patients with AAA received EVAR in our center from June 2018 to November 2019. The inclusion criteria were as follows: (1) patients 18 years or older; (2) patients diagnosed with infrarenal AAA and planned for elective EVAR on admission; (3) perioperative survival patients; (4) patients with preoperative serum albumin, total cholesterol, peripheral lymphocyte count, and other complete test results; and (5) patients with complete follow-up data. The exclusion criteria were as follows: (1) patients with certain types of AAA (rAAA and AAA involving the renal artery); (2) patients planned for open surgery or no surgery for other reasons; (3) patients with incomplete clinical data; and (4) patients with incomplete follow-up data or information collected by telephone interview.

The surgical indications for AAA mainly include the following aspects: (1) Guidelines recommend considering elective surgery for male patients with AAA diameter > 5.5 cm or female patients with AAA diameter > 5.0 cm (1, 9). Chinese experts suggest that for male patients with AAA diameter > 5.0 cm or female patients with AAA diameter > 4.5 cm, elective surgery can also be considered (21). (2) The AAA diameter grows too rapidly (>10 mm per year), early surgical treatment should be considered (22). (3) Regardless of the size of the aneurysm, if there is pain caused by the aneurysm and the possibility of rupture cannot be ruled out, timely surgical treatment is also recommended (23). (4) Surgical intervention should be considered for embolism caused by thrombus detachment in the aneurysm sac (24). (5) AAA with signs of rupture.

### Data collection

We collected patient data through an electronic medical record system and a telephone follow-up procedure. Preoperative serum albumin, lymphocyte count, and total cholesterol were collected to calculate the CONUT score. We reviewed the clinical data of the patients, including age, sex, history of aortic surgery, presence and duration of abdominal pain, comorbidities, blood pressure on admission, imaging data (AAA diameter; diameter of the proximal aneurysmal neck; length of the aneurysmal neck, distorted aneurysmal neck, calcified aneurysmal neck, mural thrombus), surgical method (whether external fenestration or branched stent technique was involved), anesthesia method, stent choice, duration of surgery, postoperative complications, intraoperative blood loss and blood transfusion, preoperative- and postoperative-related laboratory results, length of hospital stay, and total hospital cost.

### Follow-up

The follow-up time was 1 month, 3 months and 6 months, respectively. During the follow-up, aortic CTA and laboratory examination should be performed. Based on the patient’s condition during the follow-up, the aortic CTA should be repeated every 6 months to 1 year thereafter. In cases of patient loss during follow-up, the patient or his/her family was contacted by telephone to confirm the current status. Patients under surveillance at alternate medical centers were engaged through telephone for the purpose of gathering requisite data. The minimum follow-up period was 3 years after surgery, and the end point of follow-up was death.

### Clinical end points

The primary end point was mid-term mortality (duration of follow-up, >3 years). The secondary end points were surgical complications (including acute organ injury, bleeding, and ischemia–reperfusion), graft-related complications (including stent rupture, leakage, implant infection, and vessel occlusion), and reoperation. Surgical complications were based on the results of postoperative laboratory examination and clinical manifestations during hospitalization, using guidelines issued jointly by the European Society of Anesthesia and the European Society of Intensive Care Medicine (ESA/ESICM) (25). The aortic CTA should be conducted by a specialized vascular surgeon during follow-up, following the latest standards set forth by the Society for Vascular Surgery (SVS) and the European Society for Vascular Surgery (ESVS) (1, 9), to determine graft-related complications.

### Definitions

The CONUT scores were calculated from preoperative albumin concentration, lymphocyte count, and cholesterol concentration (Table 1). Patients were divided into four groups according to the CONUT score. A CONUT score of 0–1 indicated normal nutrition, 2–4 indicated mild malnutrition, 5–8 indicated moderate malnutrition, and 9–12 indicated severe malnutrition.

**Table 1:** Controlling nutritional status (CONUT) scores.

Parameter	Score			
Serum albumin, g/dL	≥3.5	3.0–3.49	2.50–2.99	<2.5
Albumin score	0	2	4	6
Total cholesterol, mg/dL	>180	140–180	100–139	<100
Cholesterol score	0	1	2	3
Lymphocytes, count/mL	≥1,600	1,200–1,599	800–1,199	<800
Lymphocyte score	0	1	2	3
Nutritional status score	0–1 (normal status)	2–4 (low risk)	5–8 (medium risk)	9–12 (severe risk)

The comorbidities of AAA have been reported elsewhere, with the main ones as hypertension, defined as a systolic blood pressure > 140 mmHg and/or a diastolic blood pressure > 90 mmHg; diabetes mellitus, defined as a history of use of insulin or other hypoglycemic drugs; stroke, defined as a history of stroke; renal dysfunction, defined as a history of kidney disease or dialysis; coronary artery disease, defined as stable angina and/or a history of coronary revascularization or myocardial infarction; and arterial disease, defined as a history of arterial surgery. Current smokers were defined as those with a history of smoking within 1 month of surgery. Aneurysm diameter; diameter of the proximal aneurysmal neck; and length of aneurysmal neck, distorted aneurysmal neck, calcified aneurysmal neck, and mural thrombus were determined by CT.

The proximal aneurysm neck was defined as the segment of the abdominal aorta between the lower edge of the renal artery and the upper edge of the aneurysm neck. Calcified aneurysmal neck was defined as the presence of an atherosclerotic plaque on CT. Distorted aneurysmal neck was defined as the angle between the first segment of the neck (the first 3 cm) and the suprarenal abdominal aorta >60 degrees. Mural thrombus was defined as thrombus adherence to the vessel wall of the aneurysm.

### Statistical analysis

Continuous variables with normal distribution were presented as mean ± standard deviation, while non-normally distributed data were reported as median (interquartile range). Categorical variables were described as the number of patients (%). Statistical comparisons involved independent and paired sample t-tests, the Mann–Whitney U test, and analysis of variance. Receiver operating characteristic (ROC) curve analysis was used to determine the optimal cut-off value for grouping. Survival analysis was conducted using Kaplan–Meier curves and log-rank tests. Cox proportional hazards models were utilized to examine the relationship between patient characteristics and mortality. Logistic regression was applied to analyze surgical complications. Variables with *p* < 0.05 in univariate analysis were included in multivariate analysis. Cox proportional hazards and logistic regression models included all baseline characteristics, relevant variables, and comorbidities to identify factors associated with mortality and postoperative complications. A *p* value <0.05 was considered statistically significant. Data analysis was carried out using SPSS 26.0 software (IBM Corp., Armonk, NY, United States).

## Results

### Screening procedure for enrolled patients

**Figure 1:**
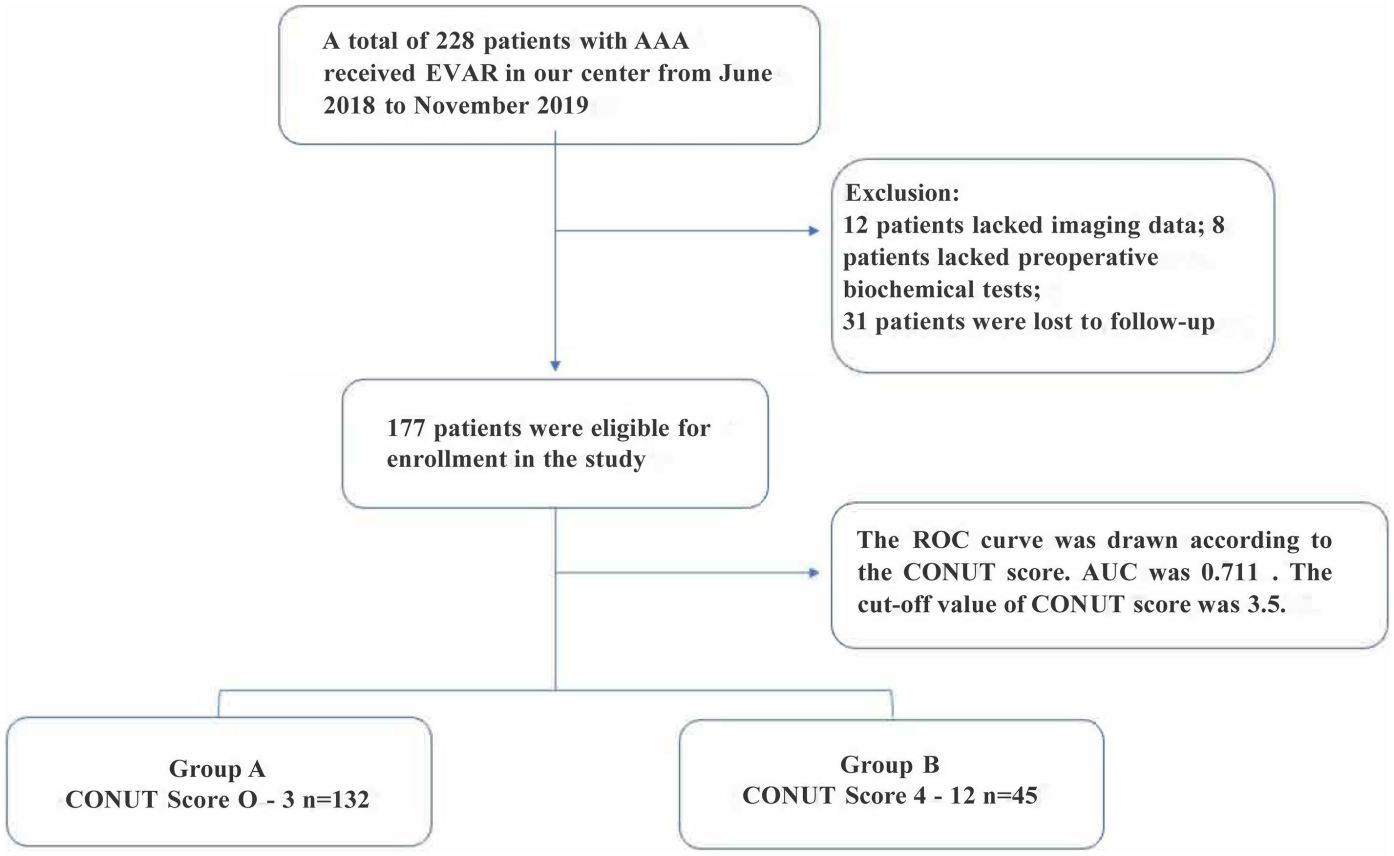
Flowchart of the patients.

**Figure 2:**
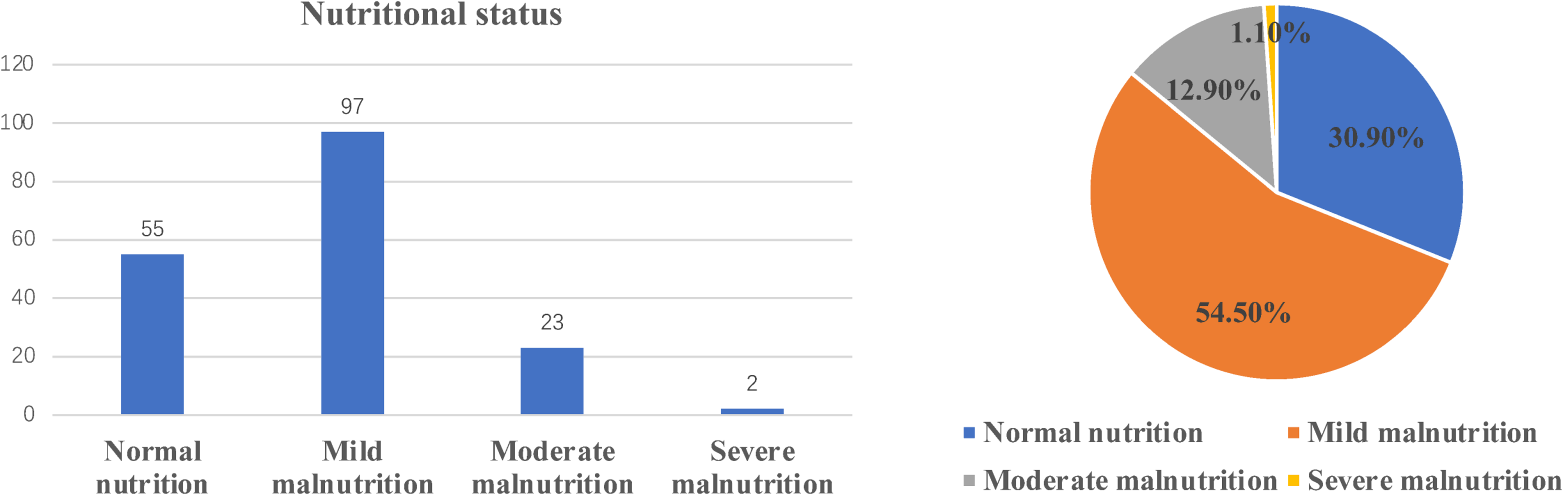
Controlled nutritional status (CONUT) scores in patients.

**Figure 3:**
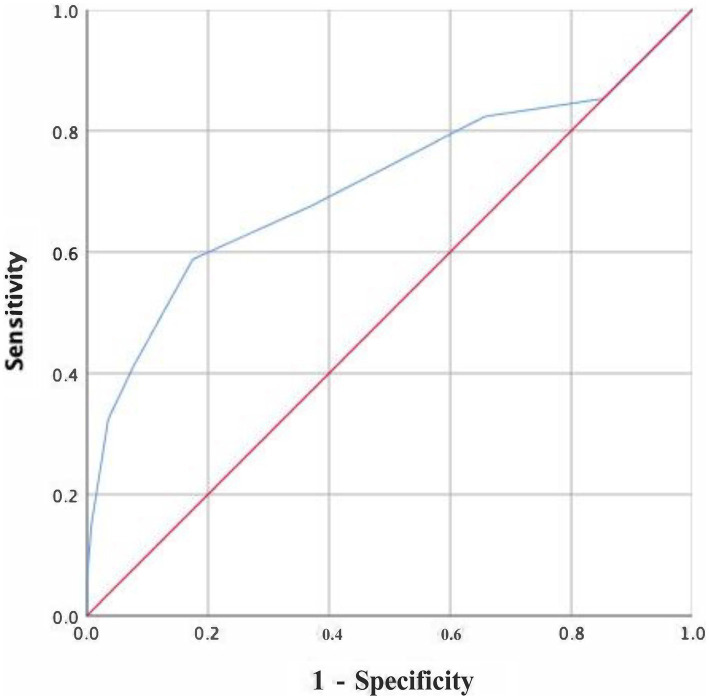
Receiver operating curve analysis for the survival rate.

From June 2018 to November 2019, a total of 228 AAA patients were diagnosed and treated in the Department of Vascular Surgery, Nanjing Drum Tower Hospital affiliated with Nanjing University. According to the inclusion and exclusion criteria, 51 patients were excluded, and a total of 177 patients were included in the analysis (Figure 1). There were 143 males (80.8%) and 34 females (17.2%). The mean age was 69. According to the CONUT criteria, the patients were classified as those with normal nutrition (*n* = 55, 30.9%), mild malnutrition (*n* = 97, 54.5%), moderate malnutrition (*n* = 23, 12.9%), and severe malnutrition (*n* = 2, 1.1%) (Figure 2). During the follow-up period, 34 patients died, and the ROC curve was drawn according to the CONUT score to predict the time of death (Figure 2). The area under the curve (AUC) was 0.711 (95% CI, 0.595–0.826, *p* < 0.001). The optimal critical value of the CONUT score grouping was 3.5, the sensitivity was 0.588, and the specificity was 0.825 (Figure 3). The study cohort comprised of 177 patients with AAA were divided into two groups according to the cut-off value: group A (CONUT score 0–3, *n* = 132) and group B (CONUT score 3–12, *n* = 45).

### Patient baseline characteristics

The patient characteristics of both groups are shown in Table 2. The median age in group A was 68.5 years (interquartile range, 64–76 years) and in group B was 72 years (interquartile range, 63–78.5 years), and there was no significant difference between the two groups (*p* = 0.257). Most patients were male (*n* = 143, 80.8%), and compared to female patients, male patients had poorer nutritional status (male vs. female, 2.72 vs. 1.65, *p* = 0.003), consistent with a previous study (19). The median CONUT score was 2 (interquartile range, 1–2) in group A and 5 (4–6) in group B (*p* < 0.001). Significant differences in serum albumin, total cholesterol, and lymphocyte count were found between the two groups (*p* < 0.001). Most patients with AAAs do not have any symptoms, and AAAs are often found by clinical screening, physical examination, or other-disease examinations. Only a few AAAs cause abdominal pain, compression, limb ischemia, rupture, and other clinical symptoms (9). In our study, 32.2% (*n* = 57) of patients had symptoms before treatment, with abdominal pain in more than 90% of these patients. In addition, most patients had varying degrees of hypertension (*n* = 130, 73.4%), and a high proportion of these patients were smokers (*n* = 111, 65.5%). However, there was no significant difference between the two groups in baseline characteristics, except for the nutritional status.

**Table 2:** Baseline characteristics of included patients.

	Group A (*n* = 132)	Group B (*n* = 45)	*p*
*Age, years*	68.5 (64–76)	72 (63–78.5)	0.257
*Sex, male/female*	103/29	40/5	0.11
*Serum albumin, mg/dL*	39.5 ± 2.6	34.5 ± 4.0	<0.001
*Total cholesterol, mg/dL*	4.3 ± 1.3	3.2 ± 0.7	<0.001
*Lymphocyte count, 10*^3^ mL	1.7 (1.3–2.1)	1.0 (0.9–1.3)	<0.001
*CONUT score*	2 (1–2)	5 (4–6)	<0.001
*CONUT grade*			
Normal	55		
Low risk	77	20	
Medium risk		23	
Severe risk		2	
*ASA classification*	2 (1.25–2.75)	2 (2–3)	0.469
*Present with symptoms*	40 (30.3%)	17 (37.8%)	0.305
*Duration of symptom (days)*	12 (3–28.5)	5 (1–24.5)	0.298
*Comorbid disease*			
Hypertension	88	32	0.582
DM	23	10	0.475
Dyslipidemia	26	8	0.778
Stroke	19	11	0.121
Renal dysfunction	38	17	0.26
Respiratory diseases	15	7	0.462
Digestive system diseases	14	6	0.618
CAD	24	12	0.222
Prior peripheral artery disease	20	8	0.677
Current smoker	94	22	0.84
*SBP, mmHg*	133 ± 15.7	135.1 ± 21.8	0.555
*DBP, mmHg*	75.7 ± 10.4	75.7 ± 11.2	0.971

CAD, coronary artery disease; CONUT, controlling nutritional status; DBP, diastolic blood pressure; DM, diabetes mellitus; SBP, systolic blood pressure. Continuous variables are presented as the mean ± standard deviation if normally distributed or the median (interquartile range) if not normally distributed. Categorical variables are presented as the number of patients (%).

### Patient examination- and surgery-related results

Table 3 shows the examination- and surgery-related results of the patients. We found no difference between the two groups in several radiographic measures of AAA, including AAA diameter; proximal aneurysmal neck diameter; and length of aneurysmal neck, distorted aneurysmal neck, calcified aneurysmal neck, and mural thrombus. Preoperative examination showed that the hemoglobin (Hb) level (*p* < 0.001), inflammation index (CRP [*p* < 0.001]), and coagulation function index [PT (*p* < 0.001), fibrinogen (*p* = 0.02)] of patients in group B were worse than those in group A, consistent with the results of previous reports (26–30). Group-B patients had higher white blood cell levels (*p* = 0.009) and lower Hb levels (*p* < 0.001) after surgery. Furthermore, the postoperative B-type natriuretic peptide level exhibited a statistically significant elevation in group B patients compared to group A patients (*p* = 0.003), suggesting a poorer postoperative cardiac function in group B, which could potentially contribute to the higher mortality rate observed in group B relative to group A. Interestingly, we found that a higher proportion of domestic stents was used in group B than in group A (*p* = 0.014); this may be related to the individual preferences of the surgeons. In addition, only a few patients received intraoperative blood transfusions, and most of them were in group B, which was mainly due to the intraoperative correction of preoperative low hemoglobin levels in group-B patients.

**Table 3:** Examination- and surgery-related results of the patients.

	Group A (*n* = 132)	Group B (*n* = 45)	*p*
*Imaging examination*			
AAA diameter, cm	5.1 ± 1.4	4.6 ± 1.2	0.158
Diameter of the proximal aneurysmal neck	2.2 ± 0.4	2.2 ± 0.3	0.881
Length of aneurysmal neck	3.8 ± 1.6	3.6 ± 1.5	0.516
Distorted aneurysmal neck	28 (21.2%)	11 (24.4%)	0.577
Calcified aneurysmal neck	83 (62.9%)	25 (55.6%)	0.477
Mural thrombus	56 (42.4%)	21 (46.7%)	0.495
*Preoperative laboratory examination*			
WBC, 10^9^/L	6.4 (5.2–7.65)	5.9 (4.48–7.73)	0.342
Hb, g/L	132 (123.5–139)	112.5 (90.25–131)	**<0.001**
Plt, 109/L	182 (146–228)	171 (139–222)	0.522
CRP, mg/L	4.7 (3.1–9)	20 (4.6–54.3)	**<0.001**
Cr, μmol/L	70.5 (61.25–87)	72 (61–98)	0.304
eGFR, mL/min	97.4 (76.78–113)	97.4 (68.1–116.4)	0.499
PT, s	11.1 (10.7–11.68)	11.7 (11.1–12.5)	**<0.001**
Fibrinogen, g/L	2.9 (2.5–3.57)	3.5 (3–4.7)	**0.02**
D-dimer, mg/L	1.64 (0.72–3.91)	2.24 (0.85–5.02)	0.242
*Methods of anesthesia*			
General anesthesia	73	29	0.284
Local anesthesia	59	16	
*Types of stent*			
Domestic stent	64	31	**0.014**
Imported stent	68	14	
*Blood loss, mL*	50 (20–150)	50 (20–200)	0.384
*Blood transfusion, mL*	0 (0–0)	0 (0–0)	**0.041**
*Operation duration, h*	1.5 (1–2.5)	1.625 (1–2.7)	0.842

AAA, abdominal aortic aneurysm; BNP, type-B natriuretic peptide; Cr, creatinine; eGFR, estimated glomerular filtration rate; Hb, hemoglobin; Plt, platelet; PT, prothrombin time; WBC, white blood cell. Continuous variables are presented as the mean ± standard deviation if normally distributed or the median (interquartile range) if not normally distributed. Categorical variables are presented as the number of patients (%). The bold values represent a statistical difference between the two groups.

### Complications and reoperations

Postoperative complications occurred in 53 patients, namely 36 patients in group A and 17 patients in group B (Table 4), and the differences were not statistically significant (*p* = 0.155). We employed the Dindo-Clavien classification to categorize postoperative complications. Mild complications were defined as Dindo-Clavien grade I and grade II, severe complications were defined as Dindo-Clavien grade III and grade IV, fatal complications were defined as Dindo-Clavien grade V. Our findings revealed that 16 cases (35.56%) in group B and 64 cases (48.48%) in group A experienced mild complications (*p* = 0.166), 5 cases (11.11%) in group B and 18 cases (13.64%) in group A experienced severe complications (*p* = 0.800), 20 cases (44.44%) in group B and 14 cases (10.61%) in group A experienced fatal complications (*p* < 0.001). These results indicated that the severity of postoperative complications was significantly greater in group B compared to group A. In group A, 19 patients had surgical complications, including 10 patients with acute organ injury (mainly acute renal insufficiency), 1 patient with postoperative incision infection, and 8 patients with other complications (including incision neuralgia, transient abnormal breathing, limb pain, and numbness), 17 patients had graft-related complications, two and five patients had type-I and-II leakage, respectively, and nine patients had postoperative stent occlusion (all of them underwent reoperation). In addition, one patient had abdominal pain 2 months after surgery and was diagnosed with implant infection. In group B, 10 patients had surgical complications, including four patients with acute organ injury, two patients with postoperative incision infection, and four patients with other complications; 10 patients had graft-related complications, four patients had type-II leakage, and six patients had postoperative stent occlusions.

**Table 4:** Patient clinical end points.

	Group A (*n* = 132)	Group B (*n* = 45)	*p*
*Follow*-*up time, months*	39 (31.5–45)	35 (13.5–42)	0.015
*In*-*hospital mortality*	1 (0.76%)	3 (6.67%)	0.085
*Midterm mortality*	14 (10.61%)	20 (44.44%)	<0.001
*Take medication as prescribed*	106 (80.30%)	31 (68.89%)	0.22
*Reoperation*	16 (12.12%)	7 (15.56%)	0.519
*Reoperation time, months*	10.56 ± 10.03	17.00 ± 17.70	0.276
*Total complications*	36 (27.27%)	17 (37.78%)	0.155
*Dindo-Clavien classification*			
*Mild complication*	64 (48.48%)	16 (31.11%)	0.166
*Sever complication*	18 (13.64%)	5 (11.11%)	0.800
*Fatal complication*	14 (10.61%)	20 (44.44%)	<0.001
*Surgical complications*	19 (%)	10 (22.22%)	0.197
Acute organ injury	10	4	
Postoperative infection	1	2	
Others	8	4	
Graft-related complications	17 (12.88%)	10 (22.22%)	0.116
Type I leakage	2	0	
Type II leakage	5	4	
Implant infection	1	0	
In-stent restenosis	9	6	
*Length of stay, days*	11.10 (8.80–14.1)	13.00 (8.85–17.60)	0.112

Table 5 presents the outcomes of both univariate and multivariate analyses assessing risk factors associated with complications, encompassing reoperation. Prior arterial disease (OR, 2.34; 95% CI, 1.024–5.348; *p* = 0.044), smoking (OR, 2.733; 95% CI, 1.300–5.748; *p* = 0.008), and D-dimer level (OR, 1.124; 95% CI, 1.011–1.250; *p* = 0.03) were risk factors, although multivariate analysis showed smoking (OR, 3.492; 95% CI, 1.426–8.553; *p* = 0.006) was an independent risk factor.

**Table 5:** Logistic regression analysis of postoperative complications and reoperations.

Variable	Univariate analysis			Multivariate analysis		
	*p*	Odds ratio	95% CI	*p*	Odds ratio	95% CI
Age	0.124	0.976	0.947–1.007			
Sex	0.464	0.744	0.337–1.642			
Disease	0.703	1.137	0.587–2.204			
CONUT score	0.222	1.11	0.939–1.311			
Present with symptoms	0.118	0.582	0.296–1.146			
Hypertension	0.314	0.692	0.339–1.416			
DM	0.979	0.989	0.434–2.254			
Dyslipidemia	0.464	0.744	0.337–1.642			
Stroke	0.673	1.198	0.518–2.770			
Renal dysfunction	0.388	0.74	0.374–1.465			
Respiratory diseases	0.2	2.1	0.675–6.535			
Digestive system diseases	0.129	0.48	0.186–1.238			
CAD	0.948	0.974	0.439–2.159			
Prior peripheral artery disease	0.044	2.34	1.024–5.348	0.449	0.632	0.193–2.073
Current smoker	0.008	2.733	1.300–5.748	0.006	3.492	1.426–8.553
Methods of anesthesia	0.065	1.89	0.960–3.718			
AAA diameter	0.628	1.075	0.802–1.440			
Diameter of the proximal aneurysmal neck	0.208	1.815	0.717–4.594			
Length of aneurysmal neck	0.616	1.062	0.839–1.345			
Distorted aneurysmal neck	0.749	0.879	0.400–1.932			
Calcified aneurysmal neck	0.445	0.756	0.368–1.551			
Mural thrombus	0.15	0.599	0.299–1.203			
Types of stents	0.38	1.341	0.696–2.583			
WBC	0.968	0.997	0.853–1.165			
Hb	0.145	0.988	0.972–1.004			
Plt	0.459	0.998	0.993–1.003			
CRP	0.064	1.009	0.999–1.018			
Cr	0.665	0.999	0.997–1.002			
eGFR	0.785	0.999	0.990–1.008			
PT	0.1	1.283	0.953–1.726			
Fibrinogen	0.089	1.316	0.959–1.806			
D-dimer	0.03	1.124	1.011–1.250	0.053	1.11	0.999–1.234

AAA, abdominal aortic aneurysm; CAD, coronary artery disease; CONUT, controlling nutritional status; Cr, creatinine; DBP, diastolic blood pressure; DM, diabetes mellitus; Hb, hemoglobin; Plt, platelet; SBP, systolic blood pressure; WBC, white blood cell.

### In-hospital and mid-term mortality

Four patients died during hospitalization. One group-A patient died of pulmonary embolism 12 h after surgery, two group-B patients died of acute postoperative hemorrhagic shock, and one group-B patient died of severe postoperative pulmonary infection with respiratory failure.

**Figure 4:**
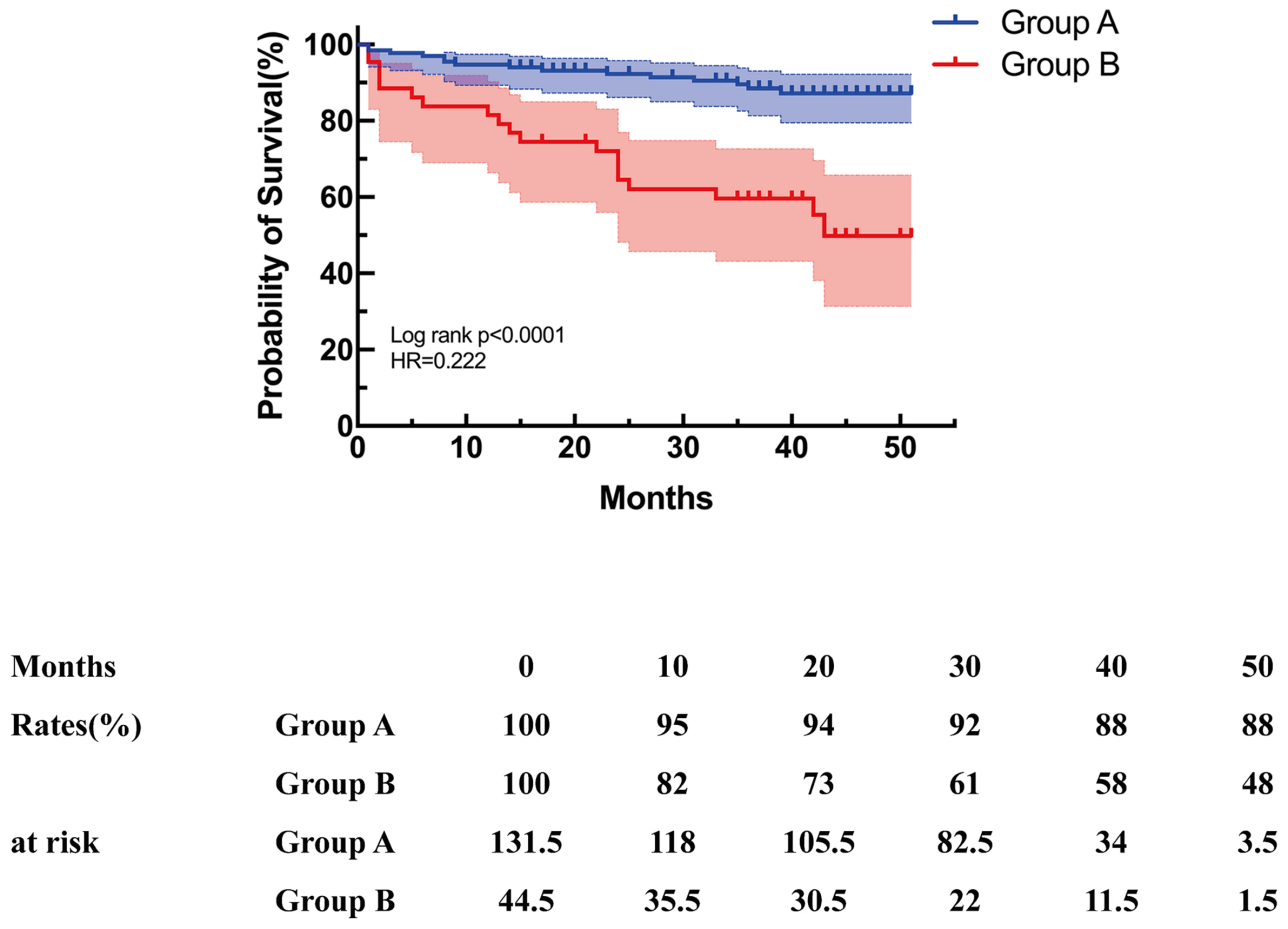
Kaplan–Meier curves for overall survival.

**Figure 5:**
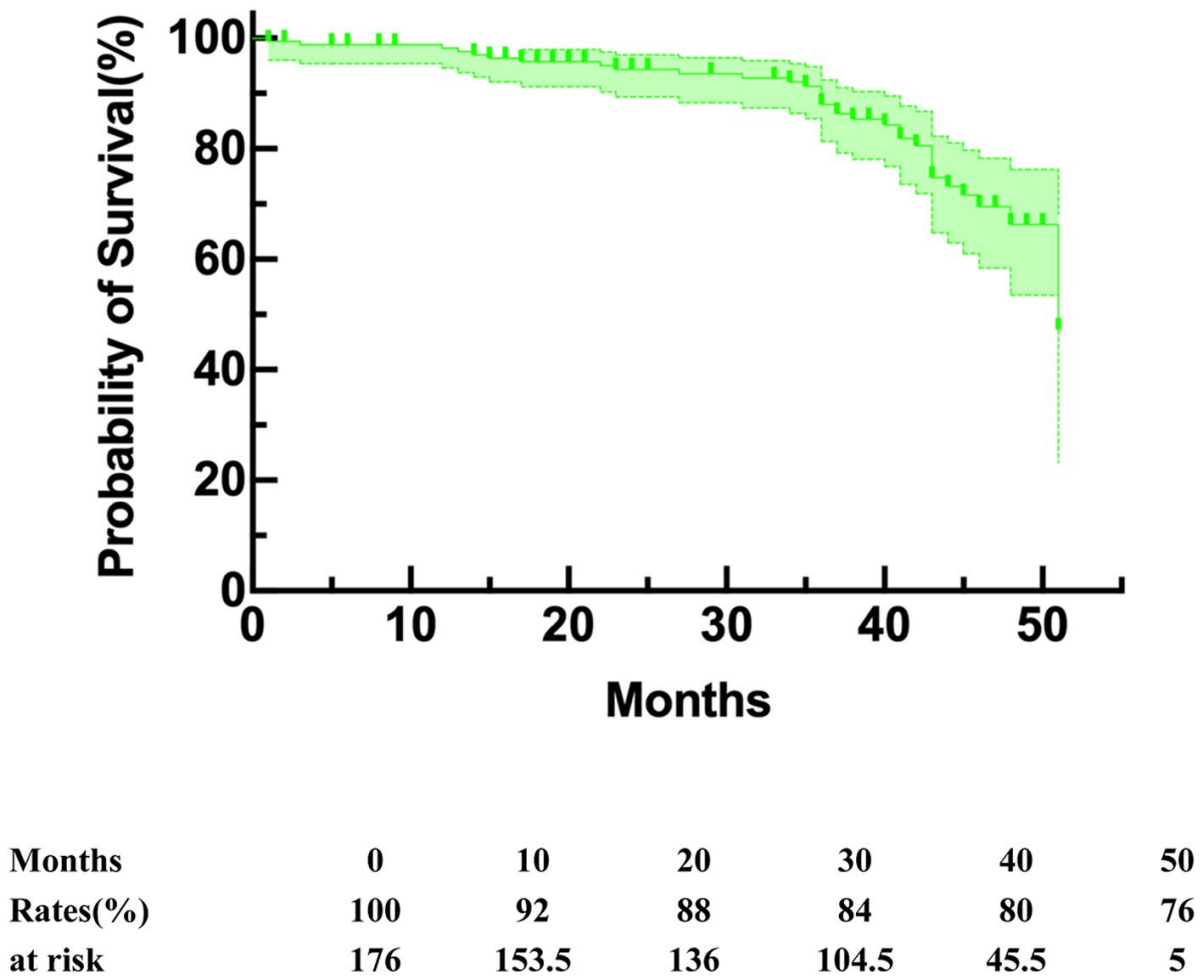
Kaplan–Meier curves for mid-term survival.

The end point of follow-up was death. The median duration of the follow-up period was 38 (interquartile range, 23–45) months. The overall survival rate during follow-up was 80.8%, with 91.5 and 81.2% at 1 year and 2 years, respectively (Figure 4). The median duration of follow-up was 39 (31.5–45) in group A and 35 (13.5–42) in group B (*p* = 0.015). Subsequent analysis revealed that group B exhibited a significantly elevated mid-term mortality rate compared to group A (*p* < 0.001). In group A, seven patients died from cancer, four from cardiovascular disease, one from cerebral hemorrhage, and one from pulmonary infection caused by aspiration during feeding due to Alzheimer’s disease. In group B, three patients died from cancer, four from cardiovascular disease, one from aneurysm rupture, two from severe pulmonary infection, two from renal failure, one from post-ERCP pancreatitis, and four patients died from underlying diseases. Kaplan–Meier survival curves were generated based on follow-up data. Figure 5 illustrates that the survival rate was notably lower in group B than in group A (log-rank test, *p* < 0.001).

The Cox proportional hazards model was employed to predict risk factors for mortality. Univariate analysis revealed that CONUT scores; respiratory diseases; stent types; preoperative Hb, preoperative CRP, preoperative PT, and preoperative fibrinogen levels were risk factors for death. Multivariate analysis confirmed that CONUT score (HR, 1.276; 95% CI, 1.029–1.584; *p* = 0.027) was an independent risk factor for mortality (Table 6).

**Table 6:** Cox regression analysis for risks of mid-term mortality.

Variable	Univariate analysis			Multivariate analysis		
	*p*	Hazard ratio	95% CI	*p*	Hazard ratio	95% CI
Age	0.528	1.011	0.977–1.046			
Sex	0.859	1.083	0.448–2.618			
CONUT score	**<0.001**	1.53	1.304–1.796	0.027	1.276	1.029–1.584
Present with symptoms	0.182	1.605	0.802–3.213			
Hypertension	0.279	0.646	0.292–1.427			
DM	0.847	0.921	0.401–2.116			
Dyslipidemia	0.081	0.518	0.248–1.084			
Stroke	0.201	1.677	0.759–3.705			
Renal dysfunction	0.128	1.693	0.860–3.333			
Respiratory diseases	**0.03**	0.416	0.188–0.920	0.062	0.396	0.150–1.048
Digestive system diseases	0.068	0.461	0.201–1.058			
CAD	0.674	0.827	0.343–1.998			
Prior peripheral artery disease	0.447	1.499	0.528–4.255			
Current smoker	0.333	0.567	0.180–1.787			
Methods of anesthesia	0.853	0.938	0.476–1.846			
AAA diameter	0.939	0.988	0.729–1.339			
Diameter of the proximal aneurysmal neck	0.859	1.096	0.399–3.006			
Length of aneurysmal neck	0.586	0.939	0.748–1.179			
Distorted aneurysmal neck	0.244	0.636	0.298–1.360			
Calcified aneurysmal neck	0.982	1.009	0.472–2.157			
Mural thrombus	0.482	1.296	0.629–2.671			
Types of stents	0.007	2.84	1.325–6.087	0.063	2.36	0.954–5.839
WBC	0.414	1.068	0.912–1.252			
Hb	<0.001	0.97	0.957–0.984	0.113	0.984	0.965–1.004
Plt	0.203	1.003	0.998–1.008			
CRP	0.001	1.01	1.004–1.015	0.221	1.006	0.997–1.015
Cr	0.11	1.001	1.000–1.002			
eGFR	0.671	0.998	0.987–1.008			
PT	<0.001	1.334	1.171–1.520	0.474	1.077	0.880–1.317
Fibrinogen	0.03	1.378	1.032–1.839	0.91	0.978	0.661–1.446
D-dimer	0.52	1.023	0.954–1.098			

AAA, abdominal aortic aneurysm; CAD, coronary artery disease; CONUT, controlling nutritional status; Cr, creatinine; DBP, diastolic blood pressure; DM, diabetes mellitus; Hb, hemoglobin; Plt, platelet; SBP, systolic blood pressure; WBC, white blood cell. The bold values represent a statistical difference between the two groups.

## Discussion

Abdominal aortic aneurysm is a fatal condition that commonly occurs during vascular surgery. Without surgical intervention, the prognosis is extremely poor. Patients often have no warning signs before rupture, and upon rupture, the risk of death is as high as 80% (8, 31). There are two kinds of surgical treatment, namely OSR and EVAR. Several randomized controlled trials in recent years have shown that EVAR is significantly better than OSR in terms of the early survival rate of AAA patients; contrary to expectations, there was no notable difference observed in long-term survival outcomes (32–34). The Society for Vascular Surgery guidelines recommend EVAR for infrarenal AAA (9). In the past, the prognostic factors of patients with AAA after EVAR were mostly focused on the morphologic and hemodynamic characteristics of AAA, and few other aspects were studied. In this study we assessed all clinical predictors of the intermediate causes of death. The CONUT score emerged as a significant independent predictor of mid-term mortality in our study (HR, 1.276; 95% CI, 1.029–1.584; *p* = 0.027).

To our knowledge, our study represents the inaugural investigation into the potential prognostic significance of nutritional status for mid-term mortality in AAA patients undergoing EVAR. Nutritional status serves as a valuable indicator of a patient’s holistic health condition, encompassing their immune response and metabolic vigor. The scoring system of the CONUT screening tool has demonstrated predictive capabilities for outcomes in various chronic diseases, malignancies, and cardiovascular conditions (11, 12, 19, 20, 35). The overall baseline nutritional status of the patients in our cohort was good. The CONUT scores indicated moderate malnutrition in 23 of 177 (12.9%) patients and severe malnutrition in 2 of 177 (1.10%) patients, with the highest recorded CONUT score of 9. We also observed a significant association between nutritional status and clinical outcomes. Using ROC curve analysis, the cut-off value of 3.5 could predict the prognosis with a specificity of 0.825 and a sensitivity of 0.588 (AUC = 0.711, *p* < 0.001) for mid-term mortality. However, Variations in the cut-off value of the CONUT score among disease models may stem from distinct nutritional statuses and pathogenic mechanisms unique to each condition.

In this retrospective analysis, we divided the cohort of patients into group A and group B based on the cut-off value. It is important to emphasize that the clinical characteristics of the two groups of patients were similar, which may have had an impact on the outcome of the patient clinical outcome. Nevertheless, in practice we found that group B patients had low Hb levels, elevated inflammatory levels, and abnormal coagulation function, which were closely related to malnutrition and discussed below. Moreover, we found that Diabetic patients often experience poor nutritional status due to gastrointestinal disorders affecting digestion and absorption, as well as nutrient loss from conditions like polyuria (36, 37). Patients with comorbidities linked to a pro-inflammatory state, such as diabetes, coronary artery disease (CAD), and renal insufficiency, may have elevated levels of pro-inflammatory cytokines, potentially exacerbating malnutrition. Inflammatory pathways activated by these comorbidities can increase metabolic demands and worsen malnutrition, potentially leading to adverse outcomes (38–40). Malnutrition can exacerbate vascular disease through chronic inflammatory responses and contribute to mortality. The CONUT score was identified as an independent predictor of mortality in the study, irrespective of comorbidities. However, other research suggests that comorbidities, such as those captured by the Charlson Comorbidity Index, can also predict mortality (41). Therefore, malnutrition and comorbidities influencing nutritional status are intertwined and collectively impact postoperative mortality.

The logistic regression analysis did not show a significant association between high CONUT scores and postoperative complications. Previous research on the correlation between CONUT scores and postoperative complications has yielded conflicting results. Kodama et al. (42) reported that CONUT scores not only predicted overall survival after open surgical repair (OSR) in AAA patients but also correlated with postoperative complications. In contrast, a study by Miyata et al. on radical hepatectomy for intrahepatic cholangiocarcinoma found that high CONUT scores were linked to poorer postoperative survival outcomes but not to postoperative complications, aligning with the current study’s findings (43). These findings underscore the complexity and variability in the relationship between CONUT scores and postoperative outcomes across different medical conditions and surgical procedures. Prior peripheral artery disease, D-dimer level, and smoking history were predictors of postoperative complications and reoperations, and multivariate analysis showed that smoking was identified as an independent predictor (OR, 3.492; 95% CI, 1.426–8.553; *p* = 0.006). Patients with prior peripheral artery disease are mostly arteriosclerosis obliterans, and they are in poor vascular health, with a high rate of postoperative in-stent restenosis and reoperation. D-dimer is an indicator of thrombosis, which may cause in-stent restenosis and require reoperation. To our best knowledge, smoking is a risk factor for thrombosis and in-stent restenosis (4, 44), and our results are consistent with this conclusion.

There are several possible explanations for the relationship between malnutrition and AAA as well as how malnutrition affected prognosis. First, malnutrition is frequently closely linked to frailty, which is characterized as a state of heightened vulnerability and functional decline (45, 46). In our study, patients in group B were older with lower Hb levels, reflecting the underlying frailty of this population. Moreover, lymphocyte count is an indicator of immune function, and patients with poor immune function tended to suffer from more comorbidities and to show a weak state. Second, inflammation is closely associated with AAA, which promotes vascular remodeling and aortic wall weakening (47), and the nutritional status reflects the extent of inflammation (27–29, 48). Nakagomi et al. (26) found that malnutrition screened by CONUT scores was significantly and positively correlated with the regulation of tumor necrosis factor α (TNF-α) and C-reactive protein (CRP) levels. Other studies have shown that proinflammatory cytokines, such as interleukin-6 (IL-6) and TNF-α, were associated with lower serum albumin concentrations (49, 50). TNF-α has the ability to promote the generation of reactive oxygen species in tissues, which in turn activates the ubiquitin-proteasome pathway, leading to the induction of muscle protein catabolism (51). TNF-α also can penetrate the blood–brain barrier, leading to anorexia (52) and further aggravating malnutrition in patients. In addition, decreased albumin levels may increase blood viscosity and activate platelets, thereby deteriorating endothelial function (53). Diehm et al. (54) found that the maximum diameter of AAA was positively correlated with the concentrations of inflammatory factors such as IL-6 and CRP. Cytokines secreted by inflammatory cells can damage tissues, causing the vessel wall to lose elasticity and rupture (55). Third, atherosclerosis is one of the pathogenic factors of AAA (4). Inflammation plays a crucial role in the advancement of atherosclerosis, and persistent chronic inflammation exacerbates malnutrition. The concept of a malnutrition-inflammation-atherosclerosis syndrome has recently emerged, highlighting a vicious cycle where malnutrition and inflammation interact, contributing to the progression of atherosclerosis and heightened cardiovascular disease-related mortality (56). Serum albumin levels are significantly negatively correlated with thiobarbituric acid-reactive substances and advanced protein oxidation products of atherosclerotic plaques, indicating that serum albumin has antioxidant effects and decreased serum albumin levels can promote atherosclerosis (57). Ishizawa et al. found that elevated serum albumin levels were associated with reduced carotid plaques and carotid intima-media thickness (58). Fourth, there is no study on the association between serum total cholesterol and cardiovascular disease prognosis, but low cholesterol has been shown to be associated with poor prognosis in a variety of cancers (59, 60). We speculate that individuals with low total cholesterol levels may exhibit more severe underlying conditions and a higher burden of comorbidities.

Interestingly, our results showed that patients in group B had longer PT values and higher fibrinogen levels than those in group A. PT mainly reflects the activities of coagulation factors I, V, VII, and X, thereby reflecting the status of exogenous coagulation system. The prolonged PT in patients with malnutrition is related to the decreased activities of the above coagulation factors. It has been reported that malnourished patients had low vitamin K levels due to inadequate intake (61), and vitamin K deficiency could lead to the low activity of the aforementioned coagulation factors and the tendency to hemorrhage. In addition, a high level of fibrinogen revealed that a large amount of fibrin is generated after coagulation, indicating that patients are prone to thromboembolic diseases. In univariate analysis, respiratory disease was a risk factor for intermediate mortality. During the follow-up period, we also found that some patients died of lung infection or lung cancer, which were mostly related to the underlying lung diseases.

The main contributions of this study were that nutritional status played an important role in the prognosis of AAA patients treated with EVAR and that CONUT scores were predictors of mid-term mortality. Patients only require relevant blood tests upon admission, which can quickly and accurately assess the nutritional status of patients, so as to provide instruction and treatment before and after surgery (such as early smoking cessation before surgery, albumin replacement therapy). In the elderly patients, it can achieve early prevention before surgery and improve the nutritional status of patients, thereby improving the survival rate of patients after surgery.

There were several limitations in this study. First, this study only included stable infrarenal AAA patients who underwent elective surgery and EVR, and patients with complex AAA and OSR were not included in this study. Further studies are needed to determine whether the health status of these patients is related to the occurrence and development of the disease and postoperative complications. Second, the CONUT scores were calculated from blood samples collected preoperatively, and they were not calculated serially, which could have changed the score over time. Third, there was selection bias in the choice of the stent, which was based on the preference of the surgeon. Fourth, the follow-up period was short. Further studies are needed to elucidate the impact of nutritional status on long-term clinical outcomes. Finally, this was a single-center retrospective study with a small sample size, and studies in different clinical settings are needed to confirm the impact of nutritional status on patient outcomes. Therefore, we advocate that more centers investigate the significance of nutritional status assessment and determine whether malnourished patients benefit from nutritional supplements.

## Conclusion

The CONUT score, a relatively new screening tool that is easy to apply in clinical practice, may play a prognostic role in the management of patients with infrarenal AAA that are treated with EVAR. Based on our results, surgeons should consider the nutritional status of AAA patients, as malnutrition may reduce the survival rate of patients after surgery. Improving the nutritional status of patients before and/or during treatment may be beneficial measure. Larger multicenter studies are needed to confirm our findings.

## Data availability statement

The original contributions presented in the study are included in the article/supplementary material, further inquiries can be directed to the corresponding authors.

## Ethics statement

The studies involving humans were approved by the Ethics Committee of Nanjing Drum Tower Hospital affiliated with Nanjing University. The studies were conducted in accordance with the local legislation and institutional requirements. The participants provided their written informed consent to participate in this study. Written informed consent was obtained from the individual(s) for the publication of any potentially identifiable images or data included in this article.

## Author contributions

S-LY: Conceptualization, Data curation, Formal analysis, Investigation, Methodology, Project administration, Software, Writing – original draft, Writing – review & editing. T-ZX: Conceptualization, Data curation, Formal analysis, Investigation, Software, Writing – original draft, Writing – review & editing. CW: Conceptualization, Data curation, Software, Writing – original draft, Writing – review & editing. KH: Conceptualization, Data curation, Writing – original draft, Writing – review & editing. X-DJ: Data curation, Writing – original draft, Writing – review & editing. TT: Data curation, Software, Writing – original draft. BS: Data curation, Software, Writing – original draft. X-LD: Conceptualization, Data curation, Formal analysis, Software, Writing – original draft, Writing – review & editing. NH: Conceptualization, Data curation, Formal analysis, Methodology, Software, Writing – original draft, Writing – review & editing. X-QL: Funding acquisition, Methodology, Resources, Supervision, Writing – original draft, Writing – review & editing.
